# Plasma concentration, cardiorespiratory and analgesic effects of ketamine-fentanyl infusion in dogs submitted to mastectomy

**DOI:** 10.1186/s12917-022-03244-1

**Published:** 2022-06-14

**Authors:** Rauane Sousa de Moura, Isabela Plazza Bittar, Janainne Hilbig Gomes, Yan Victor Rodrigues de Oliveira, Gladsthon Divino de Sousa Filho, Glauco Cézar Fragola de Faria Soares, Eliana Martins Lima, Leandro Guimarães Franco

**Affiliations:** 1 Department of Veterinary Medicine, Federal University of Goiás, 74.690-900, Goiânia, GO Brazil; 2 Development and Technological Innovation in Drugs - FarmaTec, Research Center, Universidade Federal de Goiás, Goiânia, GO Brazil

**Keywords:** Analgesia, Constant rate infusion, Mechanical nociceptive threshold, Pharmacokinetics

## Abstract

**Background:**

The analgesic and cardiorespiratory effects of ketamine, fentanyl, or ketamine-fentanyl constant rate infusion (CRI) in dogs undergoing mastectomy were evaluated. Seventeen female dogs received CRI of ketamine (GK [*n* = 6]: bolus 0.5 mg/kg; CRI 20 µg/kg/min in intra- and postoperative periods], fentanyl (GF [*n* = 5]: bolus 20 µg/kg; intraoperative CRI 5 20 µg/kg/hour and postoperative CRI 2 20 µg/kg/hour), or combination of ketamine-fentanyl (GKF [*n* = 6]: aforementioned doses) for 8 h. Cardiorespiratory, blood gas analyses, plasma drug concentrations, sedation score (SS), Pain Scores were evaluated.

**Results:**

The heart rate decreased in the GF and GKF (*p* < 0.04); the mean arterial pressure was lower in the GKF than in the GK at 35 min (*p* < 0.001). Maximum plasma concentrations were observed 5 min after bolus in the GK (2847.06 ± 2903.03 ng/mL) and GKF (2811.20 ± 1931.76 ng/mL). Plasma concentration in intraoperative period of ketamine was of > 100 ng/mL in 5/5 and 2/5 animals in the GKF and GK, respectively; and > 1.1 ng/mL of fentanyl in 4/5 and 3/5 in GKF and GF, respectively.

**Conclusion:**

Ketamine with/without fentanyl provided analgesia without significant cardiorespiratory and guaranteed the minimal plasma levels with analgesic potential during the 8 h.

## Introduction

The management of perioperative pain is a challenge in veterinary medicine, which may be associated with inadequate control in the postoperative period, culminating in the development of chronic pain and reduction of quality of life [1, 2], especially in more complex procedures, such as mastectomies, facts that have been well described in human patients [3].

The use of opioids to control pain in dogs undergoing mastectomy is relatively high and has yielded satisfactory results short term after the surgery. Within the class, advantages of fentanyl use include rapid onset [4], rapid recovery, and potent analgesic effect. Limitations of fentanyl use include opioid-induced significant respiratory depression [5, 6] and low residual analgesic effect [7], which lead to an increase in postoperative analgesic requirement [8]. The use of subanaesthetic doses of ketamine, reportedly has important analgesic properties that contribute to the modulation of central sensitization [8], contributing to the prevention of hyperalgesia [9].

In dogs, subanaesthetic doses of ketamine in CRI was considered safe [10], with better postoperative analgesia, reduced need for additional opioid administration [9], and reduced requirement for general anaesthetics [11]. The use of ketamine as a single analgesic remains controversial. In dogs undergoing mastectomy the use of the drug in low doses, and not associated with opioids, has not been able to promote effective analgesia [12], its use being recommended in association with other drugs in multimodal therapy [9]. In multimodal therapy, ketamine has been shown to decrease the requirement for analgesics in the postoperative period, and to promote long-term analgesia [13]. However, evaluation periods of such studies were limited to the immediate postoperative periods of up to 2 h. Effects of using prolonged infusions for controlling postoperative pain in late postoperative period have not yet been verified.

This study aimed to evaluate perioperative analgesic and cardiorespiratory effects of ketamine, fentanyl, or ketamine-fentanyl constant rate infusion in dogs undergoing unilateral mastectomy. We hypothesized that ketamine-fentanyl combination would promote analgesia, decreasing the need of rescue analgesic protocols, to a greater degree than would ketamine or fentanyl alone in dogs.

## Materials and methods

### Animals

Seventeen adult female client-owned dogs of different breeds weighing 11.82 ± 7.74 kg that were diagnosed with breast cancer after examination of cytology, with cancer stages I and II [14], and had surgical indication for unilateral total mastectomy were included. Animals with comorbidities, such as neoplasms from non-mammary tissue, metastasis presented in thoracic X-rays or abdominal ultrasound, abnormal laboratory test results (full blood count, creatinine, alanine aminotransferase, total protein and fractions), life-threatening arrhythmias, obesity, considered ASA Physical Status Classification ≥ III or with altered behavior, such as aggressive temperament, were excluded from the study.

### Procedure

The animals were admitted 24 h before the surgical procedure and were subjected to a 12 h food fast and a 2 h water fast. Morphine (0.5 mg/kg; intramuscularly [IM]; DIMorf; Cristália Prod. Quím. Farm. Ltda, Brazil) was administered as premedication. Fifteen minutes later, a 20 G catheter was placed in the right cephalic vein for inducing anesthesia with propofol (5.0 mg/kg; intravenously [IV]; Provine; União Química, Brazil). The dogs were intubated using a cuffed endotracheal tube which met the size of the animal, and the endotracheal tube was connected to a rebreathing system maintained with isoflurane (Isoflurane; BioChimico, Brazil) in 80% oxygen. The isoflurane vaporizer dial was adjusted to deliver a sufficient concentration for maintenance of surgical plan, based on clinical signs, including absence of palpebral reflex, absence of jaw tone, and maintenance of invasive mean arterial pressure (MAP) between 60 and 90 mmHg [11]. Animals were maintained on synchronized intermittent mandatory ventilation (SIMV) (Fabius Plus; Drägerwerk AG & Co., Germany), with the ventilator set to a minimum respiratory rate (*f*_R_) of 10 breaths/min. Subsequently, a 22 G catheter was introduced into the right dorsal pedal artery for monitoring blood pressure [systolic arterial pressure (SAP), diastolic arterial pressure (DAP), and MAP] and sampling arterial blood for blood gas and pharmacokinetic analyses.

Anaesthetic monitoring was performed using a multiparameter monitor (Datex Ohmeda; GE Healthcare, USA) and included invasive blood pressure assessment (systolic, diastolic and mean), cardiac rhythm using an electrocardiogram (lead DII, 25 mm second^−1^), oxygen saturation using a pulse oximeter (positioned on the animals’ tongue), and end-tidal partial pressure of carbon dioxide in the expired gas using a mainstream capnograph (positioned at the distal end of the endotracheal tube).

The mastectomy was performed by an experienced surgeon as described by Fossum and colleagues [15]. After the end of the surgery, the animals were hospitalized for 24. At the end of the infusion the bitches received meloxicam (0.1 mg/kg; Maxicam 0.2%; Ourofino Saúde Animal, Brazil) intravenously (IV), and after hospital discharge, dogs were prescribed meloxicam (0.1 mg/kg) for oral administration every 24 h and metamizol (25 mg/kg PO SOS).

### Study design

Animals were randomly assigned to three experimental groups, using with a software (Microsoft Excel 2007), depending on the IV treatment protocol: (1) ketamine (Ketamine Agener; União Química, Brazil) [group ketamine (GK) (*n* = 6): bolus 0.5 mg/kg; intra- and postoperative CRI 20 µg/kg/min], (2) fentanyl (Fentanest; Cristália Prod. Quím. Farm. Ltda, Brazil) [group fentanyl (GF) (*n* = 5): bolus 5 µg/kg; intraoperative CRI 5 µg/kg^/^hour and postoperative CRI 2 µg/kg^/^hour], or (3) ketamine-fentanyl [group ketamine and fentanyl (GKF) (*n* = 6): aforementioned doses].

The CRI loading dose was administered 5 min before skin incision. Intraoperative CRI started immediately after bolus administration and ended after animal’s extubation. A new solution containing the doses previously mentioned for each group was prepared for postoperative CRI, which started after extubation and was administered during the following 8 h. All infusions were administered using a peristaltic infusion pump (SR670; Samtronic Industria e Comercio LTDA, Brazil). The drugs were diluted in a standardized manner in 0.9% saline solution until a final volume of 10 mL was obtained (infusion rate of 10 mL/h). To prepare the infusion, the volumes of the drugs calculated from the established doses were initially added to a 10 mL syringe, and then the syringe was filled in its entirety with a volume of saline solution. The infusions were prepared by a professional who was not involved in the evaluations; evaluators were blinded to the treatments until the end of the experimental phase.

### Data collection

Evaluations of cardiorespiratory parameters and collect of blood samples were conducted at the following time points: preanaesthetic period [baseline (0) and 10 min after premedication]; intraoperative period (5 min after anaesthesia induction and subsequently every 10 min until a total of 75 min); and postoperative period (one, two, four, six, eight, 12, and 24 h after anaesthetic recovery, which started after extubation). The intraoperative period lasted 75 min for all the animals in order to minimize bias caused by different periods of CRI administration.

The cardiorespiratory variables evaluated were as follows: heart rate (HR); respiratory rate (*f*_R_); non-invasive SAP in the preoperative and postoperative periods; invasive DAP, SAP, and MAP in the intraoperative period; pulse oximetry (SpO_2_); and end-tidal carbon dioxide (FE^´^CO_2_) in the intraoperative period. The apnea was defined as the synchronization of the animal's *f*_R_ with the ventilator frequency (10 breaths/min), and its occurrence was documented.

Arterial blood samples were collected, and blood gas analyses were performed every 20 min in the intraoperative period and every 4 h in postoperative period. The samples were obtained from the catheter with a heparinized 1 mL blood gas syringe (BD A-Line; Becton, Dickinson and Company, USA) (Roche Cobas B121; Roche Diagnóstica Brasil Ltda., Brasil).

To evaluate the plasma concentration of ketamine and fentanyl, arterial blood samples were collected from the dorsal pedal artery and placed in tubes containing lytic heparin. Samples from 14 animals, five animals in each group (GK and GKF) and four animal in GF were used for the analysis. allocated to the GK, GKF (5/6), and GF (4/5), respectively, were used for the analysis.

The samples were subsequently centrifuged at 1800 g, and the plasma was separated and stored at − 20 ºC. Plasma concentrations of ketamine and fentanyl were determined using coupled high-performance liquid chromatography in a triple quadrupole mass spectrometer (HPLC–MS/MS), according to the methodology previously described by Toki et al. (2019) [16] and Huynh et al. (2005) [17]. The system consisted of an Agilent 1200 chromatograph equipped with an Agilent XRS Pursuit C18 column (particle size 5 µm; 150 × 4.0 mm) hyphenated by a Sciex API 3200 mass spectrometer and a computer with Analyst software; a mobile phase composed of a mixture of acetonitrile and 0.1% formic acid in water in the proportion 75:25 in the isocratic regimen was passed through the column at a constant flow of 1.0 mL/min. The drugs were monitored in positive ionization mode (ESI^+^); ion ratios (m/Z) of ketamine [M + H^+^] and fentanyl [M + H^+^] are 238.189/125.00 and 337.350/105.100, respectively. Plasma samples were thawed and 125 µL aliquots were pipetted to Eppendorf tubes; 25 µL of propranolol (500.00 ng/mL; internal standard) and 25 µL of 0.1 M sodium hydroxide were added to the sample of 1 mL of the extraction mixture (MTBE: 80:20 dichloromethane). The samples were centrifuged for 5 min at 8000 g, and 700 µL of the supernatant was transferred to a test tube. After drying at 40 °C with dry air at a constant flow of 25 kgf/m^2^, the samples were reconstituted with 250 µL of the mobile phase and 10 µL was injected into the HPLC–MS/MS system. The samples were analyzed against calibration curves prepared using white canine plasma contaminated with known concentrations of ketamine (0.10 − 5000.00 ng/mL) and fentanyl (0.10 − 150.00 ng/mL) with R^2^ of 0.99 and calculated weight of 1/X^2^.

Postoperative pain scores were evaluated according to the short version of Glasgow Composite Measure Pain Score (GCPS) [18] and mechanical nociceptive threshold (MNT), after the end of the surgical procedure and extubation every 15 min in the first hour, after that every 120 min for up to 12 h, and 7 and 14 days.

The short form of GCPS includes 6 behavioral categories with associated descriptive expressions of vocalization, attention to wound, mobility, response to touch, demeanor, and posture/activity. The assessment of MNT was carried out with a Von Frey digital algometer (digital analgesimeter; INSIGHT®, Brazil). The evaluation area was the 1 − 3 cm region around the surgical wound, which was evaluated at 6 different points (two points in regions thoracic, abdominal, and inguinal on the right and left sides) and averaged.

Postanaesthetic sedation was assessed according to the scores developed by Bergadano and colleagues, (2009) [19], throughout the postoperative period. Behavioral changes throughout the infusion period were also recorded, such as increasing locomotor activity, head and neck stereotypical movements, twitching, sialorrhea, tearing and emesis.

### Rescue analgesia

If the postoperative pain scores were > 5 points (5/20), morphine (0.1 mg/kg IV) was administered, and the animal was reassessed after 10 min of administration; if necessary, a new dose was administered. The number of analgesic rescues was documented throughout the assessment.

In the interval between the 12 and 24 h evaluations, the analgesic evaluation using GCPS was performed every 4 h to guarantee adequate analgesia to the animal in the interval among the evaluations. If there was a need to anticipate the analgesic rescue, the data obtained were considered for the time point 24 h, and the exact time of administration of the rescue was presented descriptively.

### Statistical analysis

Power test was performed using statistics software (SigmaPlot for Windows) for a sample size calculation. The normality of data distribution was checked using Shapiro–Wilk Test. Statistical analyses were performed using GraphPad Prism 8 (GraphPad Software Inc., CA, USA) for Windows. The data related to HR, f_R_, arterial pressure, FE^´^CO_2_, SpO_2_, plasma concentration, and MNT are presented as means ± standard deviations and subjected to analysis of variance for repeated measures, followed by Tukey’s test, for comparison among the time points within each group and among groups at each time point. To evaluate sedation, and analgesia using the Glasgow Scale the Kruskal–Wallis test was used, followed by the Dunn’s test for comparison among time points within the same group and among groups at each time point. A significance level of 5% (*P* ≤ 0.05) was set as the threshold.

## Results

A total of 58 dogs were screened for enrolment in the study, but only 18 met the inclusion criteria. A female dog from the GF was withdrawn for the study after data collection because she presented complications during the surgical procedure that required another surgical intervention.

The mean expired isoflurane fractions during anesthesia were 1.52 V%, 0.98 V%, and 1.2 V% in the GK, GKF, and GF, respectively.

There was no difference in the number of analgesic rescues among the groups studied. It was observed that analgesic rescues occurred later in the GF than in the GK and GKF. Requirements for analgesic rescues were verified up to 6 h in the GK and GKF and only from 6 h in the GF. In the GK, one animal (1/6) received rescue at 15 min and another at 2 h; in the GKF, two animals (2/6) received rescue at 2 h; in the GF, one (1/5) animal received rescue at 6 h and another at 8 h.

### Cardiorespiratory variables

The results corresponding to cardiorespiratory parameters in the intraoperative and postoperative period are shown in Tables 1 and 2.

**Table 1:** Mean ± standard deviation values of cardiorespiratory parameters, in dogs treated with ketamine (GK: bolus 0.5 mg kg^−1^; CRI 20 µg kg^−1^ min^−1^ in intra- and postoperative periods), fentanyl (GF: bolus 5 µg kg^−1^; intraoperative CRI 5 µg kg^−1^ h^−1^ and postoperative CRI 2 µg kg^−1^ h^−1^), or combination of ketamine-fentanyl (GKF: aforementioned doses) in intraoperative period

		**Anaesthesia (minutes)**								
**Variable**	**Group**	**Baseline**	**5**	**15**	**25**	**35**	**45**	**55**	**65**	**75**
HR (beats minute^−1^)	GK	104 ± 13	129 ± 27	126 ± 32^a^	120 ± 42^a^	123 ± 33^a^	117 ± 22^a^	120 ± 16^a^	113 ± 16^a^	115 ± 13^a^
	GKF	110 ± 22	87 ± 9	71 ± 8^#b^	69 ± 6^#b^	72 ± 16^#b^	66 ± 14^#b^	72 ± 24^#b^	75 ± 16^#b^	82 ± 16^#b^
	GF	97 ± 35	113 ± 32	88 ± 25^ab^	57 ± 15^#b^	58 ± 10^#a.b^	71 ± 13^b^	68 ± 15^#b^	66 ± 11^#b^	69 ± 12^#b^
*f*_R_ (breaths minute^−1^)	GK	30 ± 10	17 ± 1^#^	17 ± 1^#^	16 ± 1^#^	15 ± 1^#^	15 ± 1^#^	16 ± 2^#^	16 ± 2^#^	16 ± 2^#^
	GKF	34 ± 14	17 ± 2^#^	16 ± 1^#^	15 ± 2^#^	15 ± 1^#^	15 ± 1^#^	14 ± 3^#^	15 ± 1^#^	14 ± 3^#^
	GF	28 ± 4^#^	20 ± 5^#^	14 ± 2	15 ± 3^#^	15 ± 4^#^	15 ± 2^#^	17 ± 2^#^	17 ± 1^#^	17 ± 1^#^
MAP (mmHg)	GK	-	75 ± 14	70 ± 16	74 ± 13	90 ± 8^a^	81 ± 13	78 ± 10	77 ± 11	77 ± 9
	GKF	-	74 ± 7	72 ± 9	60 ± 14	66 ± 10^b^	72 ± 12	76 ± 15	77 ± 6	79 ± 9
	GF	-	73 ± 6	75 ± 8	73 ± 2	81 ± 15^ab^	89 ± 20	65 ± 40	87 ± 25	74 ± 10
SpO_2_ (%)	GK	-	99 ± 2	99 ± 2	99 ± 2	99 ± 1	99 ± 1	99 ± 1	99 ± 1	99 ± 2
	GKF	-	98 ± 1	98 ± 1	97 ± 2	97 ± 2	97 ± 2	97 ± 1	97 ± 1	98 ± 1
	GF	-	99 ± 1	99 ± 1	99 ± 1	99 ± 1	98 ± 2	98 ± 1	98 ± 1	98 ± 1
FE^´^CO2 (mmHg)	GK	-	36 ± 6	35 ± 6	34 ± 3	35 ± 4	34 ± 2	33 ± 5	34 ± 5	34 ± 5
	GKF	-	27 ± 6	31 ± 1	31 ± 1	30 ± 3	32 ± 4	31 ± 2	32 ± 4	32 ± 5
	GF	-	28 ± 2	30 ± 3	31 ± 4	33 ± 5	32 ± 4	33 ± 5	32 ± 5	31 ± 5

^a^^, b, c^ Significant difference between group. ^#^Significant difference in treatment with a basal time point. *HR* Heart rate, *f*R respiratory rate, *MAP* mean arterial pressure, *SpO*_*2*_ pulse oximetry, *FE’CO2* end-tidal carbon dioxide

**Table 2:** Mean ± standard deviation values of cardiorespiratory parameters, in dogs treated with ketamine (GK: bolus 0.5 mg kg^−1^; CRI 20 µg kg^−1^ min^−1^ in intra- and postoperative periods), fentanyl (GF: bolus 5 µg kg^−1^; intraoperative CRI 5 µg kg^−1^ h^−1^ and postoperative CRI 2 µg kg^−1^ h^−1^), or combination of ketamine-fentanyl (GKF: aforementioned doses) in postoperative period

		Postoperative period (hours)								
**Variable**	**Group**	**Baseline**	**0.25**	**0.5**	**1**	**2**	**4**	**6**	**8**	**12**
HR (beats minute^−1^)	GK	104 ± 13	107 ± 14^a^	96 ± 20	87 ± 10^a#^	95 ± 14^a^	86 ± 13	86 ± 13	98 ± 39	101 ± 14
	GKF	110 ± 22	104 ± 13^a^	97 ± 9	89 ± 7^a^	87 ± 14^a.b#^	90 ± 13	90 ± 7	95 ± 14	94 ± 16
	GF	97 ± 35	82 ± 24^b^	71 ± 13	70 ± 20^b#^	75 ± 21^#^	65 ± 17^#^	79 ± 14	83 ± 18	83 ± 17
*f*_R_ (breaths minute^−1^)	GK	30 ± 10	40 ± 23	36 ± 15	43 ± 12^#^	47 ± 11^#^	37 ± 18	32 ± 10	30 ± 10	43 ± 47
	GKF	34 ± 14	44 ± 41	39 ± 23	45 ± 23	52 ± 39	35 ± 13	36 ± 11	29 ± 10	35 ± 15
	GF	28 ± 4	54 ± 21	44 ± 25	68 ± 37	43 ± 27	29 ± 17	33 ± 20	38 ± 19	27 ± 7
SAP mmHg	GK	136 ± 25	137 ± 9^a.b^	139 ± 12	136 ± 15	136 ± 24	148 ± 17	144 ± 17	140 ± 22	143 ± 16
	GKF	153 ± 13	153 ± 15^a^	155 ± 18	151 ± 17	170 ± 28	160 ± 15	160 ± 21	151 ± 12	143 ± 21
	GF	136 ± 13	132 ± 23^b^	163 ± 12	148 ± 8	138 ± 35	136 ± 15	141 ± 10	158 ± 7^#^	147 ± 3

^a^^, b, c^ Significant difference between group. ^#^ Significant difference in treatment with a basal time point. *HR* Heart rate, *f*R respiratory rate, *SAP* systolic arterial pressure

The arterial blood gas results are shown in Table 3.

**Table 3:** Mean ± standard deviation values of arterial blood gas analyses in dogs treated with ketamine (GK: bolus 0.5 mg kg^−1^; CRI 20 µg kg^−1^ min^−1^ in intra- and postoperative periods), fentanyl (GF: bolus 5 µg kg^−1^; intraoperative CRI 5 µg kg^−1^ h^−1^ and postoperative CRI 2 µg kg^−1^ h^−1^), or combination of ketamine-fentanyl (GKF: aforementioned doses) in intraoperative and postoperative period

		**Anesthesia (minutes)**				**Postoperative (hours)**		
**Variable**	**Group**	**20**	**40**	**60**	**80**	**1**	**4**	**8**
pH	GK	7.30 ± 0.08	7.29 ± 0.07	7.29 ± 0.06	7.31 ± 0.06	7.33 ± 0.04	7.39 ± 0.02	7.38 ± 0.04
	GKF	7.36 ± 0.06	7.33 ± 0.06	7.31 ± 0.05	7.27 ± 0.04^#^	7.34 ± 0.02	7.38 ± 0.03	7.41 ± 0.01
	GF	7.29 ± 0.08	7.27 ± 0.04^#^	7.27 ± 0.03^#^	7.25 ± 0.02^#^	7.33 ± 0.01	7.37 ± 0.02	7.41 ± 0.01
PaO_2_ (mmHg)	GK	294.6 ± 89.2	319.8 ± 80.2	304.4 ± 88.0	293.5 ± 4.4	80.4 ± 3.0	90.8 ± 13.9	83.7 ± 7.9
	GKF	300.9 ± 62.3	328.1 ± 106.2	296.2 ± 89.8	290.3 ± 89.8	78.2 ± 6.6	79.4 ± 5.6	80.7 ± 6.3
	GF	291.5 ± 4.07	274.2 ± 36.1	266.5 ± 29.0	282.7 ± 88.9	80.8 ± 3.1^#^	84.3 ± 5.0^#^	84.2 ± 5.4^#^
PaCO_2_ (mmHg)	GK	45.9 ± 11.2	46.4 ± 10.2	45.2 ± 8.5	42.9 ± 6.3	40.9 ± 4.2	31.4 ± 1.7	32.3 ± 1.5
	GKF	38.7 ± 6.1	40.6 ± 6.9	43.0 ± 5.8	48.5 ± 8.5^#^	43.3 ± 4.3	36.3 ± 2.0	33.1 ± 2.1
	GF	44.2 ± 15.3	44.6 ± 8.8	43.3 ± 6.9	46.8 ± 5.3	41.5 ± 2.2	34.3 ± 3.9	35.3 ± 1.1
HCO_3_^−^ (mmol L^−1^)	GK	21.8 ± 1.8	21.3 ± 1.3	20.8 ± 1.5	20.9 ± 0.8	21.1 ± 2.0	18.6 ± 0.4	18.6 ± 0.7
	GKF	21.0 ± 0.5	20.9 ± 0.7	20.8 ± 0.8	21.8 ± 1.7	23.2 ± 1.2	21.1 ± 1.2	20.3 ± 1.2
	GF	7.30 ± 0.08	20.8 ± 3.0	20.1 ± 2.5	20.6 ± 2.4	21.0 ± 0.7	19.7 ± 2.6	21.2 ± 0.3

^#^ Significant difference in treatment with a basal time point. pH; arterial partial pressure of oxygen (PaCO_2_); arterial partial pressure of carbon dioxide (PaCO_2_); arterial bicarbonate (HCO_3_^−^)

### Pharmacokinetic parameters

There was no difference in the plasma concentration of ketamine and fentanyl, maximum concentration, and clearance among the groups studied (Table 4).

**Table 4:** Mean values ± standard deviation of the pharmacokinetic parameters of ketamine and fentanyl in plasma samples of dogs treated with ketamine (GK: bolus 0.5 mg kg^−1^; CRI 20 µg kg^−1^ min^−1^ in intra- and postoperative periods), fentanyl (GF: bolus 5 µg kg^−1^; intraoperative CRI 5 µg kg^−1^ h^−1^ and postoperative CRI 2 µg kg^−1^ h^−1^), or combination of ketamine-fentanyl (GKF: aforementioned doses)

	**Group**	**AUC**	**C**_**max**_** (ng mL**^**−1**^**)**	**Cl (mL minute**^**−1**^** kg**^**−1**^**)**
**KET**	GK	3854.55 ± 1698.62	2894.62 ± 2860.39	0.034 ± 0.034
	GKF	8686.15 ± 7290.39	2217.34 ± 1927.83	0.024 ± 0.018
**FENT**	GKF	7.84 ± 2.53	3.9 ± 0.81	0.041 ± 0.026
	GF	7.45 ± 4.12	5.3 ± 1.77	0.063 ± 0.048

Area under the curve (AUC); clearance (Cl); maximum plasma concentration (C_max_)

**Fig. 1:**
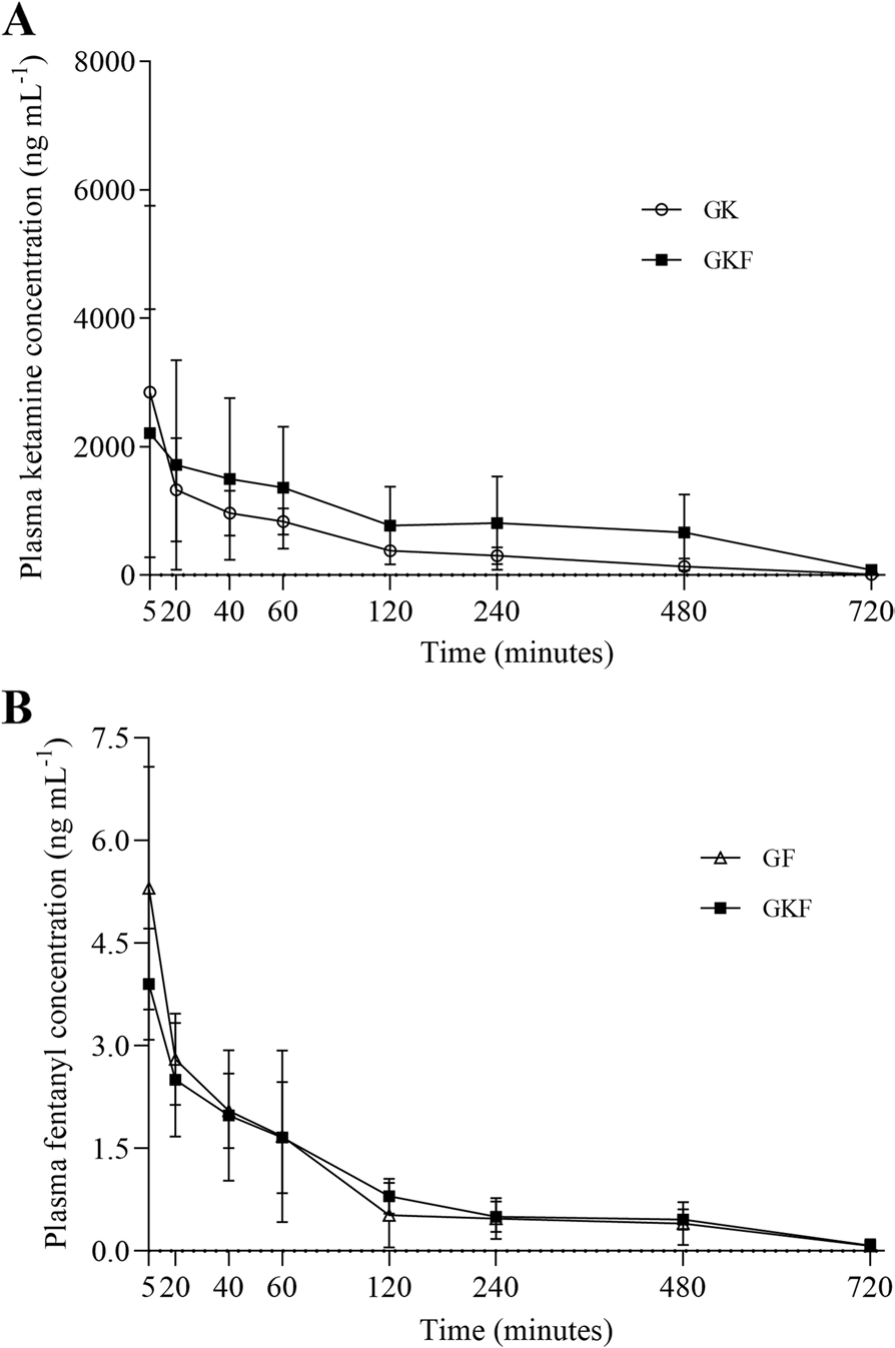
Mean ± standard deviation of plasma concentration (ng mL^−1^) of ketamine (graph **A**) e fentanyl (graph **B**) obtained from fourteen dogs undergoing unilateral mastectomy treated for 8 h with ketamine (GK: bolus 0.5 mg kg^−1^; CRI 20 µg kg^−1^ min^−1^ in intra- and postoperative periods), fentanyl (GF: bolus 5 µg kg^−1^; intraoperative CRI 5 µg kg^−1^ h^−1^ and postoperative CRI 2 µg kg^−1^ h^−1^), or combination of ketamine-fentanyl (GKF: aforementioned doses) in intra- and postoperative periods

The maximum observed ketamine concentration 5 min after the bolus administration in the GK and GKF were 2847.06 ± 2903.03 and 2811.20 ± 1931.76 ng/mL, respectively (Fig. 1). The postoperative ketamine infusion was sufficient to maintain plasma a concentrations of > 100 ng/mL in all animals treated with fentanyl (5/5) and only in two animals treated exclusively with ketamine (2/5).

An infusion of 5 µg/kg^/^hour of fentanyl during the operation was sufficient to maintain plasma levels of > 1.1 ng/mL in 4/5 animals in the GKF (2.51 ± 1.18) and 3/4 animals in the GF (2.96 ± 1.79). The increase of ketamine in the GKF did not significantly changes the plasma concentration of fentanyl during the intraoperative period, compared to the GF.

### Sedation and analgesia

**Fig. 2:**
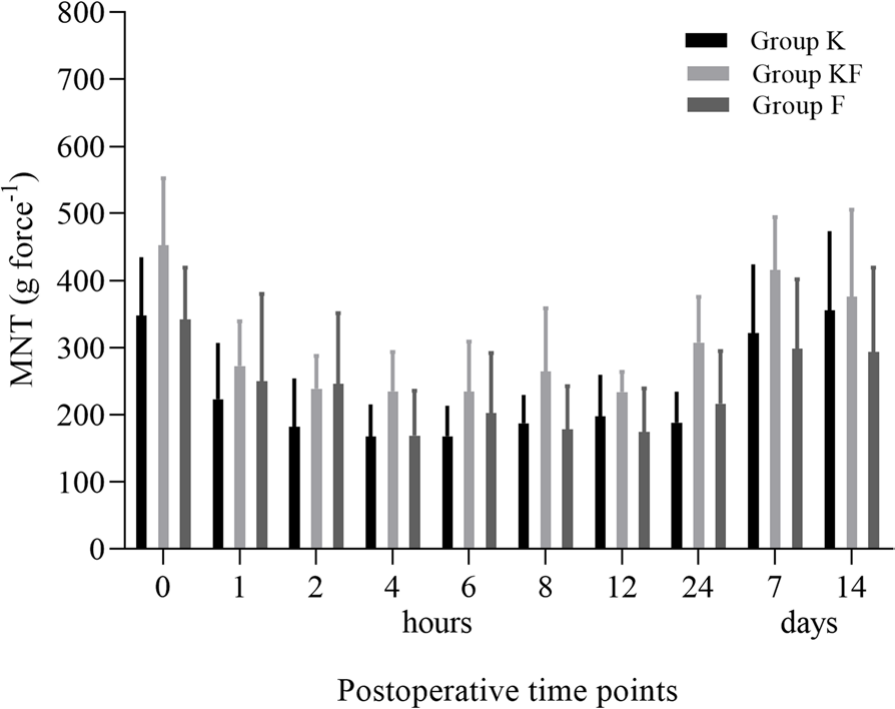
Mean ± standard deviation of mechanical nociceptive thresholds (MNT, g force^−1^) in seventeen dogs undergoing unilateral mastectomy treated for 8 h with ketamine (GK: bolus 0.5 mg kg^−1^; CRI 20 µg kg^−1^ min^−1^ in intra- and postoperative periods), fentanyl (GF: bolus 5 µg kg^−1^; intraoperative CRI 5 µg kg^−1^ h^−1^ and postoperative CRI 2 µg kg^−1^ h^−1^), or combination of ketamine-fentanyl (GKF: aforementioned doses) in postoperative time points up to 14 days

The results related to MNT are shown in Fig. 2. Regardless of the group evaluated, a significant reduction in MNT up to 12 h after the operation (*P* < 0.03) was observed; at T24h it remained significantly lower than at baseline in groups GK (*P* = 0.03) and GKF (*P* = 0.02). MNT values returned to baseline level in all groups on postoperative day 7. Higher sedation scores were found at time points between 15 and 30 min than at baseline in the GKF (*P* < 0.02), GK (*P* < 0.009), and GF (*P* < 0.04).

**Fig. 3:**
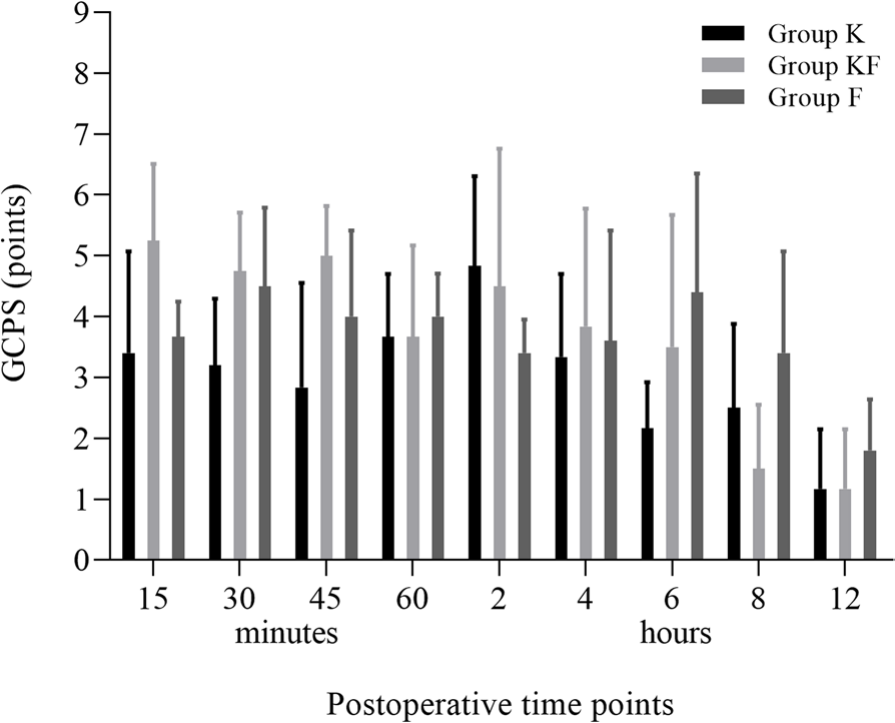
Mean ± standard deviation of Glasgow Composite Measure Pain Score (GCPS, number escale) in seventeen dogs undergoing unilateral mastectomy treated for 8 h with ketamine (GK: bolus 0.5 mg kg^−1^; CRI 20 µg kg^−1^ min^−1^ in intra- and postoperative periods), fentanyl (GF: bolus 5 µg kg^−1^; intraoperative CRI 5 µg kg^−1^ h^−1^ and postoperative CRI 2 µg kg^−1^ h^−1^), or combination of ketamine-fentanyl (GKF: aforementioned doses) in postoperative time points up to 12 h

There were no significant differences in pain scores recorded with GCPS among groups (Fig. 3).

The following psychomimetic changes were observed: GF: vocalization in 1/5 animals; GK: sialorrhea in 3/6 animals and vocalization in 1/6 animals; and GKF: vocalization in 1/6 animals.

## Discussion

The main findings of this study were as follows: a) The infusions did not trigger significant cardiovascular changes in any of the groups throughout the experimental phases; b) all groups experienced satisfactory analgesia during the intraoperative period; c) there was no significant differences between groups regarding postoperative analgesia during the 24-h period; d) fentanyl and ketamine doses used did not promote a steady state, but guaranteed minimal plasma levels with potential for analgesia during the 8 h of administration.

The reduction in HR observed in the groups treated with fentanyl was already expected, considering that the action on µ opioid receptors inhibits voltage-dependent calcium channels, reducing neurotransmission by cardiac vagal neurons [20]. Although a reduction in HR was observed, this did not trigger significant hemodynamic repercussions even during the perianaesthetic period.

The ketamine-fentanyl association did not culminate in the sympathetic stimulation expected by the use of ketamine [10, 13]. However, when infused alone, the drug contributed to the maintenance of HR at a level close to the baseline levels during the infusion period. This effect is due to the activation of adrenergic receptors and the alteration of the calcium flow to the intracellular medium with positive inotropism, along with a reduction in the bradycardia-inducing effect of fentanyl. The sympathetic effects of ketamine such as increasing HR have been demonstrated by infusions delivering doses of ≥ 40 µg/kg^/^hour in awake dogs [10].

Despite the reduction in HR observed over the transoperative period in the GF, the reduction in MAP was only observed at 35 min of anesthesia compared to the GK. MAP was maintained at > 60 mmHg without the use of vasoactive drugs in any of the animals, possibly due to a lower requirement for isoflurane, attributed to the analgesic effect of both premedication and infusions [11, 21].

The absence of significant changes in blood gas values during surgery demonstrated the effectiveness of the ventilation mode and the adjustment adopted for the study. Although respiratory depression attributed to fentanyl has already been described for infusions with a dose of 4 µg/kg/hour [5], which is close to the dose used in our study (2 − 5 µg/kg/hour), others authors did not observe any ventilatory changes that could be recognized in the blood gas analysis [4, 7]. It was not possible to state that the treatment with fentanyl did not promote respiratory depression depending on the ventilatory support used; however, the presence of apnea in dogs treated with fentanyl, as verified by the synchronization with the ventilator, demonstrates the need for ventilatory support in the intraoperative period.

A preliminary study published by our group demonstrated that a dose of 20 µg/kg/min was safe [10]; the dose of ketamine used in the infusion in this study was based on these findings. Doses of 10 µg/kg/min have been reported in previous studies with controversial results related to analgesia [19, 22], and in a study conducted by Sarrau and colleagues [22], this dose was considered to be unable to promote adequate analgesia. These described results supported the use of a dose of 20 µg/kg/min.

The doses of ketamine used in this study were not able to promote a steady state during the infusion; it was possible to observe large variations in concentrations among individuals throughout the evaluated time points. The highest plasma concentration was observed in the first minute of infusion because of the bolus administration. Even at high doses of infusion, such as 30 and 50 µg/kg/min [13], the steady state was not observed after the bolus administration. This lack of steady state can be explained by the fast clearance of the drug; therefore, high infusion doses for a prolonged period are required for at least five half-lives [13].

The large variations in the maximum plasma concentrations observed among individuals and among studies are justified by the differences in doses [19], intraspecific differences [23], high drug clearance rate [13], and the variations related to biotransformation by cytochrome P450 [24]. Ketamine is biotransformed by the cytochrome P450 enzyme family, mainly by the CYP2B6, CYP2C9, and CYP3A4 isoforms [24, 25].

To our knowledge, no previous study conducted pharmacokinetic evaluation of ketamine associated with fentanyl and evaluated the influence of one drug on the plasma concentrations and the clearance rate of the other. The individual plasma concentrations of ketamine in the GKF were higher than those in the GK throughout the infusion period. The GKF was able to maintain a plasma levels above 160 ng/mL for up to 8 h in all animals (5/5), whereas the GK showed plasma levels of > 110 ng/mL in only one animal (1/5).

The CYP3A4 isoform is responsible for the biotransformation of fentanyl [26]. It is believed that the adoption of fentanyl in the protocol resulted in an increase in plasmatic concentrations, probably through alteration of ketamine metabolism and biotransformation. Fentanyl, by reducing HR, can also alter cardiac output, thereby altering the clearance and redistribution of ketamine [27, 28].

Plasma fentanyl concentrations showed a peak 5 min after the bolus administration, which decreased gradually over the infusion period. Similar ketamine infusions, fentanyl infusions showed no steady state throughout the infusion. The minimum effective analgesic range for fentanyl was reported to be 0.95 − 2.00 ng/mL for dogs and humans [29–32]. The plasma fentanyl concentrations observed in our study were maintained within analgesic levels for up to one hour, with the decrease coinciding with the reduction in the infusion rate in the postoperative period. The infusion rate was reduced after the end of anesthesia to decrease the cumulative effect and consequent respiratory depression, commonly observed with the use of µ agonist opioids [5, 6], and because of the absence of studies showing the cardiovascular and respiratory impact of prolonged fentanyl infusion.

Regardless of the treatment, the drugs were not sufficient to maintain the nociceptive threshold close to baseline levels in the first 12 h. However, GCPS indicated that the animals were not showing signs of significant pain. Postoperative pain induced by mastectomy is mainly caused by the inflammatory response to surgical trauma, by peripheral sensitization; when not treated correctly, it can lead to the activation of long-term stimulation mechanisms, with consequent hyperalgesia [33].

Although the scale used has not been validated for evaluating mastectomy-related pain, the results, obtained by an experienced evaluator, indicated that the animals remained comfortable in the postoperative period; however, superiority of the treatments could not be evaluated. The reduction of MNT demonstrated that the drugs used in the treatments were not able to prevent peripheral sensitization, which is normally prevented with the use of drugs of other classes that prevent the conduction of primary afferent sensory fibers or have an anti-inflammatory action.

Ketamine is known to reduce acute pain in the postoperative period [3], but evidence suggesting its long-term benefits and a decreased in the requirement of opioid analgesics is still limited [3, 8]. Although the superiority of analgesia with increased ketamine to fentanyl compared to its isolated use was not evident, the results corresponding to the GK were sufficient to demonstrate that ketamine infusion shows analgesic effect without the association with opioid as indicated by the plasma concentrations. In dogs, use of ketamine in multimodal infusion protocols [13] or as a bolus [9, 34] demonstrated a decrease in MNT at the surgical site in dogs after surgical procedure [9, 34], as well as in GCPS scores [9], thus demonstrating a decrease in central sensitization with an improvement in intra- and postoperative analgesia, but without decreasing peripheral sensibilization.

Fentanyl bolus administration allows a decrease in MNT for a short time [35], whereas a CRI regimen was shown to increase the thermal thresholds for up to 300 min during the infusion [36]. However more studies are warranted to conclude the effect of continuous infusion on nociceptive mechanical threshold.

The treatments contributed to reduce postoperative pain and contributing to the recovery of the animals, as indicated by the MNT values with return to baseline values in all animals on the 14^th^ day of assessment.

The limitations of this study were the characteristics of a clinical study, in which variables related to an individual are large. To minimize these impacts, the selection of animals was judicious, which led to a reduction in the number of animals/group and may have reduced the magnitude of differences among treatments, especially regarding pharmacokinetic behavior. Another issue that may have influenced the demonstration of differences among treatments regarding MNT, is related to the use of NSAIDs after the end of the infusion. The authors believe that despite the limitations, the results presented are significant.

## Conclusion

The infusion of ketamine, alone or in combination with fentanyl, could promote satisfactory analgesia in the intra and postoperative periods, and the doses used did not cause cardiorespiratory changes requiring cessation of its use in the postoperative period for up to 8 h. All infusions led to the maintenance of analgesic plasma concentrations, but the addition of fentanyl to ketamine treatment promoted an increase in plasma ketamine concentration. The treatments tested are suitable protocols to be considered for balanced anaesthesia during unilateral total mastectomy. Further studies are required to determine the impact of one drug on the other's biotransformation.

## Data Availability

The datasets used and/or analysed during the current study are available from the corresponding author on reasonable request.

## Abbreviations

CRI: Constant rate infusion
GK: Treatment group ketamine
GF: Treatment group fentanyl
GKF: Treatment group ketamine-fentanyl
ASA: American Society of Anesthesiologists
IM: Intramuscularly
IV: Intravenously
MAP: Mean arterial pressure
SIMV: Synchronized intermittent mandatory ventilation
*f*_R_: Respiratory rate
SAP: Systolic arterial pressure
DAP: Diastolic arterial pressure
SpO_2_: Pulse oximetry
HR: Heart rate
FE^´^CO_2_: End-tidal carbon dioxide
GCPS: Glasgow Composite Measure Pain Score
MNT: Mechanical nociceptive threshold
